# Building a professionally recognised clinical trial workforce: Is it time for an education and accreditation strategy?

**DOI:** 10.1177/17407745251328287

**Published:** 2025-03-27

**Authors:** Simone Spark, Prudence Perry, Thobekile Mthethwa-Pitt, Dragan Ilic, Anne Woollett, Sophia Zoungas, Marina Skiba

**Affiliations:** 1 School of Public Health and Preventive Medicine, Monash University, Melbourne, VIC, Australia; 2 Monash University Clinical Trial Centre, Faculty of Medicine, Nursing and Health Sciences, Monash University, Melbourne, VIC, Australia; 3 TrialHub, Alfred Health, Melbourne, VIC, Australia

**Keywords:** Clinical trials, clinical trialist, discipline-specific education, accreditation, allied health profession

## Abstract

Evidence-based medicine relies heavily on well-conducted clinical trials. Australia lacks a discipline-specific education pathway to provide the specialist skills necessary to conduct clinical trials to the highest standards. Unlike allied health professionals, clinical trialists who currently possess the specialist skills to conduct clinical trials do not receive professional recognition. The National Health and Medical Research Council defines ‘clinical trialist’ to include site staff as well as investigators. In this perspective piece, we explore the importance of discipline-specific education in creating a job-ready workforce of clinical trialists; the need for recognition of clinical trialists as an allied health profession in concert with their existing medical, nursing and other professional qualifications and outline a proposed specialist education and accreditation strategy.

## The value of clinical trialists

Clinical trials are an important component of evidence-based medicine, providing knowledge about diseases and their management and informing decisions about treatments and patient safety.^1^ A ‘clinical trial’ is a sub-set of health research that prospectively assigns human participants, or groups of humans, to one or more intervention(s) to evaluate the effects on health-related outcomes.^2–5^ Unlike other forms of health research, clinical trials are able to determine if new interventions are effective and safe. Clinical trials are enormously diverse, ranging from early phase to post-marketing trials, involving healthy volunteers to critically ill participants, assessing a range of interventions including drugs, devices and lifestyle changes and using a variety of trial designs, from traditional to innovative.^6^ In addition to assessing the efficacy and safety of new interventions, and providing an opportunity to explore prophylactic and preventive interventions, the Australian Commission on Health and Safety in Healthcare’s National Clinical Trial Governance Framework (the Framework) stipulates that all Australians should have access to clinical trials as a treatment option.^2^ While all trials need to comply with local and international guidelines, how these guidelines are operationalised varies depending on the unique requirements of each trial.

When discussing the clinical trial workforce, guidelines and reference documents refer to investigators only.^2,7–9^ The National Health and Medical Research Council Competencies for Australian Academic Clinical Trialists (NHMRC Competencies)^10^ defines the term ‘clinical trialist’ (Table 1) to include clinical trial site staff as well as investigators. This definition acknowledges a diverse workforce (including non-clinical staff such as data managers) that together ensures trials are conducted appropriately and ethically, protecting the safety of trial participants and producing credible results that enable the public to have confidence in clinical trial findings which support the practice of evidence-based medicine.

**Table 1.** Clinical trialists.^10^

**Site staff:** All staff involved in a trial, with a particular emphasis on the role of the Clinical Research Co-ordinator **Site Principal Investigator (PI):** The PI at a given site, who may or may not be the trial designer **Lead Principal Investigator:** The PI who has designed the clinical trial and who takes overall responsibility for it

The workforce of today (and of yesterday) has little awareness of clinical research as a career option or clinical trialist as a job role.^11^ Those who do aspire to become a clinical trialist find the way forward challenging at best due to the lack of discipline-specific education pathways. Unlike other professions within healthcare where entry and progression within the profession are clear,^11^ this career trajectory is undefined.

With the growth and advancement of the Australian clinical trial landscape,^5,6,12^ there is an increasing need for a job-ready workforce that has the expertise to understand, interpret and implement relevant clinical trial guidance/standards and adapt specific requirements to varying research settings. The current lack of discipline-specific educational pathways and benchmarked qualifications^11,13^ to become a clinical trialist, as defined by the NHMRC Competencies,^10^ is a barrier to the appropriate growth of professional practice within the clinical trial sector, and also to the much-needed professional recognition of clinical trialists.

## How can education and training support clinical trialists?

Clinical trialists should be qualified by education, training and experience.^7,14^ However, there is limited reference to what health discipline(s), qualifications and/or experience are applicable, thus leaving this unaided and somewhat opaque determination to the Principal Investigator and the Human Research Ethics Committee. The recent endorsement of the Framework^2^ now shares this responsibility for determining appropriate education and training with the organisation where the research is being conducted. Despite this important development, a lack of defined discipline-specific education pathways remains, with sponsors, Human Research Ethics Committees and the Framework relying almost entirely on the mandated International Council for Harmonisation (ICH) Good Clinical Practice (GCP)^7^ certification as an indicator of adequate clinical trial-specific education and training. ICH GCP certification ensures that clinical trials are designed, recorded and reported in an ethically and scientifically sound way. TransCelerate accreditation^15^ standardises GCP training ensuring that all accredited training includes the minimum criteria to sufficiently cover ICH GCP. While GCP training is critical, it falls short of providing the underpinning theoretical knowledge upon which clinical trials are predicated,^16,17^ leaving entry-level staff without the in-depth understanding needed to conduct an expanding range of clinical trials in accordance with requirements for quality, safety and integrity.

The pressure on the clinical trial workforce to expand, with efficient upskilling to match, is further compounded by the increasing complexity of clinical trials and the diversification of trial designs.^11,18^ Discipline-specific education that is benchmarked within the Australian Qualifications Framework (AQF)^19^ will provide a consistent knowledge and skills development framework. This will also reduce the risk to trial quality and, more importantly, the risk to participant safety.

The absence of discipline-specific benchmarked education has left on-the-job training as a key upskilling option.^20^ Consequently, many employers try to compensate for the lack of discipline-specific education by requiring experience (usually a minimum of 2 years) from job applicants. This route has its limitations, as experience is difficult to come by without first obtaining employment.^11^ In addition, the range of applicants for any role in clinical trials will vary from new graduates through to those with postdoctoral qualifications, irrespective of discipline background.^21,22^ Discipline-specific education, upskilling and professional development are therefore often facilitated through on-the-job training, which adds strain to clinical trial teams who have variable teaching and mentorship capabilities.^23^ The resultant inconsistences in knowledge development across the workforce could be negated with the introduction of discipline-specific benchmarked education.

**Figure 1.**
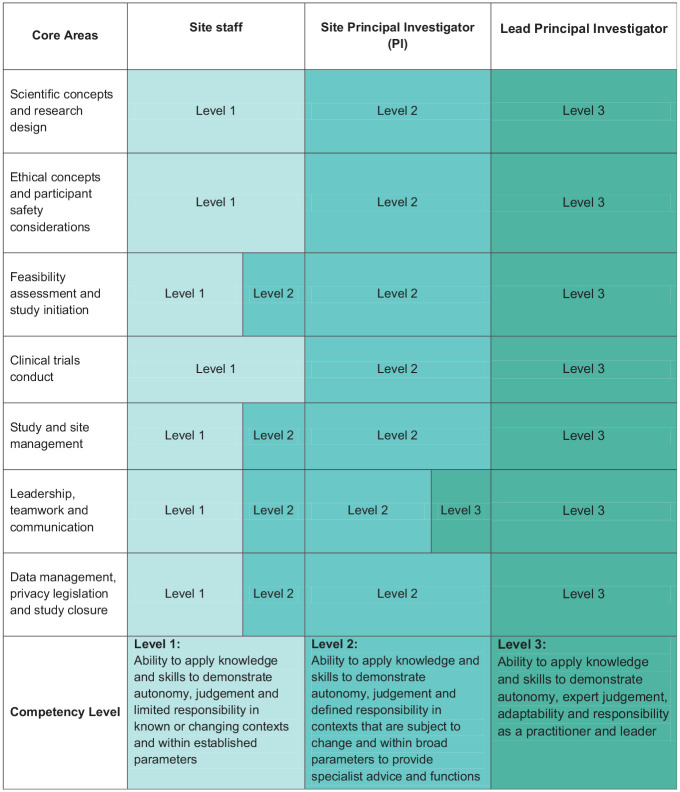
NHMRC core areas and competency levels for clinical trialists. NHMRC: National Health and Medical Research Council.

The NHMRC Competencies^10^ stipulate mastery of 7 core discipline areas at 3 competency levels underpinning clinical trial conduct for trial staff, investigators and principal investigators, respectively (Figure 1). The NHMRC intends that these core areas and levels of competency should be incorporated into higher education. We contend that these competencies form the basis of accredited entry-level education for all clinical trialists. This education would complement ad hoc support from the valuable, non-standardised clinical trial training currently available, organisation-specific onboarding, and other local competency upskilling and mentorship.

## Is it time for clinical trialists to be recognised as an allied health profession under Allied Health Professions Australia?

Experimental interventions, particularly when administered therapeutically, are not without risk, making it imperative that the consequences of a poorly conducted trial are avoided. The conduct of clinical trials requires specialist skills and knowledge of why clinical trials need to be well conducted, as well as an understanding that trialists have a responsibility to ensure reliable, high-quality outcomes. Clinical trialists provide participants with quality care by ensuring they can enter and participate in a trial safely, by ensuring tests and other trial specific assessments are performed appropriately, and by prioritising participant health during the study. When clinical trialists lack understanding of critical aspects of trial conduct, such as why entry criteria are important, why following the protocol is critical, why documenting deviations matter, when to withdraw a participant from a trial and the implications of releasing erroneous results, the consequences can be very serious, not only to individual participants^24^ and industry sponsors^25^ but to the community as a whole.^26^ Furthermore, there is an expectation across both the healthcare sector and the community that people delivering any form of intervention in patient care have a minimum level of education, experience and currency.^27^

The specialist skills exhibited by clinical trialists, over and above their skills as doctors, nurses or other professionals, warrant recognition as a profession in their own right. As clinical trialists generate the evidence that underpins prevention, diagnosis and treatment of medical conditions, it stands to reason that professional recognition of clinical trialists could be achieved with accreditation as an allied health profession. Accreditation would mean that, for the first time, clinical trialists would be required to have certain discipline-specific benchmarked education, as well as maintain professional development and adherence to standards.

In addition to upholding standards, an accreditation system also provides a body with the power to cancel accreditation if a clinical trialist fails to meet the necessary requirements or engages in unacceptable behaviour. Currently, the Australian Code for the Responsible Conduct of Research (the Code)^28^ provides guidance for institutions needing to investigate breaches of the Code. However, if the research does not involve public funding, the Code is only ‘encouraged’.^28^ Furthermore, when instances of poor research conduct have been identified, an institution’s recourse generally does not extend beyond terminating employment as they have no obligation, or pathway, to warn future employers of the individual’s behaviour. Only in extreme circumstances, where there is an indication of harm to individuals or the public, would criminal charges be an option.

Australia is not alone in identifying the need for professional recognition of clinical trialists with the United States calling for similar initiatives^11^ and the United Kingdom launching their UK Clinical Trials Talent Taskforce in August 2023.^29^ Both the International Accrediting Organization for Clinical Research^30^ and the World Health Organization^31^ are also calling for specialist, fit-for-purpose, accreditation and training.

A lack of professional recognition for clinical trialists, underpinned by appropriate education and required standards, has led to heterogeneity and non-uniformity of clinical trial job titles, roles, responsibilities and site structures, which can risk misrepresentation of speciality skills. In other words, individuals working in the trial space may be providing specialist care without the necessary skills.

Accreditation could cover all clinical trialists including, but not limited to, academic clinical trialists working on investigator-initiated research and clinical trialists conducting industry-sponsored clinical trials. This form of accreditation would facilitate appropriate levels of quality and safety in clinical trials across Australia while awarding clinical trialists with long sought professional standing.

Allied health professionals in Australia are currently co-ordinated by Allied Health Professions Australia (AHPA),^32^ a nationally recognised body that accredits and advocates for allied health professionals. AHPA currently registers a number of professionals (e.g. arts therapists, speech pathologists), who offer vastly different types of care, but all with the same level of professional accountability as those conducting clinical trials.^32^ AHPA’s membership consists of peak bodies/member organisations, that is, organisations that represent specific allied health professions (similar to Health and Care Professions Council in the United Kingdom). Each of these bodies/organisations outlines the requirements individuals must meet in order to receive and maintain accreditation within that specific allied health profession. This includes minimum qualifications, required hours of practice, annual professional development and, in some cases, stipulating the guidelines/standards that allied health professionals are expected to follow. AHPA does not require consistency in the terminology the peak bodies/member organisations use and examples include registered, accredited, credentialed and certified^32^ with ‘accredited/accreditation’ being the term of preference in the current discussion. We suggest that, subsequent to formal qualifications and/or relevant experience, clinical trialists could be recognised through accreditation with AHPA.

Allied health professional accreditation as a clinical trialist would not overwrite, supersede or make redundant any other accreditation or registration the clinical trialist may hold. Rather, like many other allied health professionals, accredited clinical trialists could hold dual professional registration, enabling them to gain recognition and practice in both disciplines. This approach will require adherence to professional standards, registration requirements and guidelines for both the clinical trialist discipline and their foundational specialty field, including ongoing continuing education.

There are few candidate bodies who have standing in the clinical trial space at a national level who may represent a suitable peak body/member organisation in the future. Like many other professions, this body/organisation may impose a fee as part of accreditation. It is premature to outline exactly what this would look like but it is anticipated that, like other allied health professions, this would not be onerous. Peak bodies/member organisations of AHPA may be self-regulated or may be regulated through the National Registration and Accreditation Scheme.

AHPA is being proposed initially for consideration, however, as conversations advance in this important topic, potentially other societies, organisations and so on may evolve to offer accreditation to individuals with a diverse range of backgrounds and qualification who are working as clinical trialists.

## Where to next?

In Australia, the AQF is the national policy for regulated qualification in education and training. AHPA^32^ requires a minimum AQF 7 tertiary-level (equivalent to a bachelor’s degree) education for all Allied Health Professionals.^32^ The discipline specialisation which aligns the NHMRC Competencies and industry-standard entry-level practice can be achieved through a mix of pathways including education and training, or recognition of prior service. Discipline-specific education for clinical trialists could consist of a graduate qualification, such as a Graduate Certificate or a Graduate Diploma (both AQF 8) in clinical trials, or an undergraduate qualification, such as a bachelor’s degree (AQF 7) in a health-related discipline with a major in clinical trials. Such educational programmes should include curricular topics as outlined in the NHMRC Competencies (Figure 1). A combination of continued practice in clinical trials (a minimum number of annual hours working on clinical trials) along with continuing professional development (minimum education activities designed to ensure current competencies in clinical trials) would then be required to maintain professional accreditation.

To take the next step, we advocate for (1) accredited benchmarked, discipline-specific qualifications for tomorrow’s clinical trialists and (2) professional recognition for all clinical trialists, through membership with an accrediting peak body/member organisation. Such innovations would provide current and aspiring clinical trialists with a pathway that can open the door for recognition of their profession. The authors hope that this article will stimulate discussion which will lead to a consensus that formalises a process of accrediting the clinical trial profession.
